# The genetics of neuroticism: Insights from the Maudsley rat model and human studies

**DOI:** 10.1017/pen.2023.4

**Published:** 2023-08-04

**Authors:** David A. Blizard, Nelson Adams, Dorret I. Boomsma

**Affiliations:** 1 Department of Biobehavioral Health, Pennsylvania State University, University Park, PA, USA; 2 Department of Psychological Sciences, Winston Salem State University, North Carolina, USA; 3 Netherlands Twin Register, Department of Biological Psychology, Vrije Universiteit Amsterdam, Amsterdam, The Netherlands

**Keywords:** Neuroticism (N), Irritable bowel syndrome (IBS), Genetics, Animal models, Maudsley rat strains, Heritability, GWAS, Stress

## Abstract

We examine some of the genetic features of neuroticism (N) taking as an animal model the Maudsley Reactive (MR) and Maudsley Nonreactive (MNR) rat strains which were selectively bred, respectively, for high and low open-field defecation (OFD) starting in the late 1950s. To draw analogies with human genetic studies, we explore the genetic correlation of N with irritable bowel syndrome (IBS). We review progress with the rat model and developments in the field of human complex trait genetics, including genetic association studies that relate to current understanding of the genetics of N. The widespread differences in the tone of the peripheral sympathetic nervous system that have been found between the Maudsley strains, particularly those observed in the colon, may underly the differences in OFD (MNR, higher sympathetic tone and zero defecation). In humans, a large genome-wide association study (GWAS) reported six genes contributing to IBS, four of which were implicated in mood and anxiety disorders or were expressed in the brain, with three of the four also expressed in the nerve fibers and ganglia of the gut. Heritability of N is estimated at around 50% in twin and family studies, and GWASs identified hundreds of loci, enabling estimation of genome-wide correlations (r_g_) with other traits. Significantly, the estimate for r_g_ between risk of IBS, anxiety, N, and depression was >0.5 and suggested genetic pleiotropy without evidence for causal mechanisms. Findings on the adrenergic pharmacology of the colon, coupled with new understanding of the role of the locus ceruleus in modifying afferent information from this organ, generate hypotheses that challenge traditional cause/effect notions about the relationship of the central nervous system to peripheral events in response to stress, suggest specific targets for gene action in the Maudsley model and emphasize the value of reciprocal evaluation of genetic architecture underlying N in rodents and humans.

Modeling a human personality dimension from a behavior genetic perspective in 1960 was a challenging endeavor as twin and family studies tended to be small, molecular approaches were not yet possible, and at the phenotypic level trait dimensions in personality were incompletely defined and their psychophysiological foundation only starting to emerge. In this review, we discuss progress toward genetic modeling of neuroticism (N), a major trait to emerge in nearly all descriptions and theories of personality, whether they are based on a lexical approach (Franic, Borsboom, Dolan, & Boomsma, 2014) or developed more from a biological perspective such as Eysenck’s three-factor model of personality (Eysenck, 1967) assessed by the Eysenck Personality Questionnaire (EPQ), Gray’s reinforcement sensitivity theory (Gray & McNaughton, 2000), or Cloninger’s four-dimensional personality model (Cloninger, Svakic, & Przybeck, 1993).

Eysenck’s formulation of personality in the 1950s contributed to the development of the Maudsley strains as an animal model of N, which was sometimes referred to as emotionality at the time. While the term “emotionality” was used more broadly in animal models, “neuroticism” gained further adherence in human literature, particularly after its adoption by the Big Five model of personality. In accordance with recent trends and the general idea that N reflects a type of negative emotionality (Widiger & Oltmanns, 2017), we will use the term N with occasional reference to emotionality in animal studies.

The selection of a rat model for exploration of the human personality dimension of N was guided by pragmatic considerations: as an omnivorous mammal, this species possesses many physiological and neurological systems in common with humans, especially those that influence the autonomic nervous system; an enormous literature, dating back more than 100 years, documents its physiology and behavior; it is a species whose husbandry is understood and for which institutional resources have been developed. Creating a genetic model enabled other researchers across different disciplines to focus relatively easily on the same biological variations by breeding the strains in their own laboratories.

Undoubtedly, there is a wide range of techniques at our disposal to investigate the involvement of genes in the various facets of human personality. Of particular interest is the N trait, largely due to its robust link to anxiety, depression, and other psychiatric disorders (Widiger & Oltmanns, 2017). Reviews by de Castro Gomes et al. (2013) and Sartori, Landgraf, and Singewald (2011) illustrate, respectively, in rats and mice, the diversity of models available for study of anxiety and stress-related behaviors. In some cases, selection has been applied to behavioral variation in tests of timidity (Fujita, Annen, & Kitaoka, 1994); in others, the discovery of differences in a key test between existing inbred strains has led to those strains being proposed as a relevant model. Tests involving response to electric shock, such as active avoidance conditioning, have also been used to differentiate strains and develop relevant models (Fernandez-Teruel et al., 2021). With the development of methods to manipulate single genes in mice, models have also been created to focus on the role of specific genes in the behavior in question.

One advantage of the Maudsley model, shared with some other strains (Fernandez-Teruel et al., 2021; Fujita et al., 1994), is the existence of a large database, comparing the strains on diverse biobehavioral traits. An important disadvantage, when considering use of most, if not all, rat models is their lack of availability from commercial sources.

In rodent studies, emotionality is often indexed by open-field activity and open-field defecation (OFD), and gene-mapping studies confirmed the genetic correlation (Blizard & Bailey, 1979) between the two (Turri, Datta, DeFries, Henderson, & Flint, 2001). Broadhurst (1960) opted for an index based on OFD, which putatively reflected both central nervous system (CNS) and peripheral physiological function and successfully established the Maudsley Reactive (MR) and Nonreactive (MNR) rat strains, which showed a clear separation after only a few generations of selection (Figure 1). Here, we first describe the development of these strains followed by distinctions between them that are relevant to current work. We then move to discuss human genetic studies of N and the genetic associations of N with irritable bowel syndrome (IBS). We finish by highlighting how recent studies may provide a context for new applications of the Maudsley strains.

**Figure 1.**
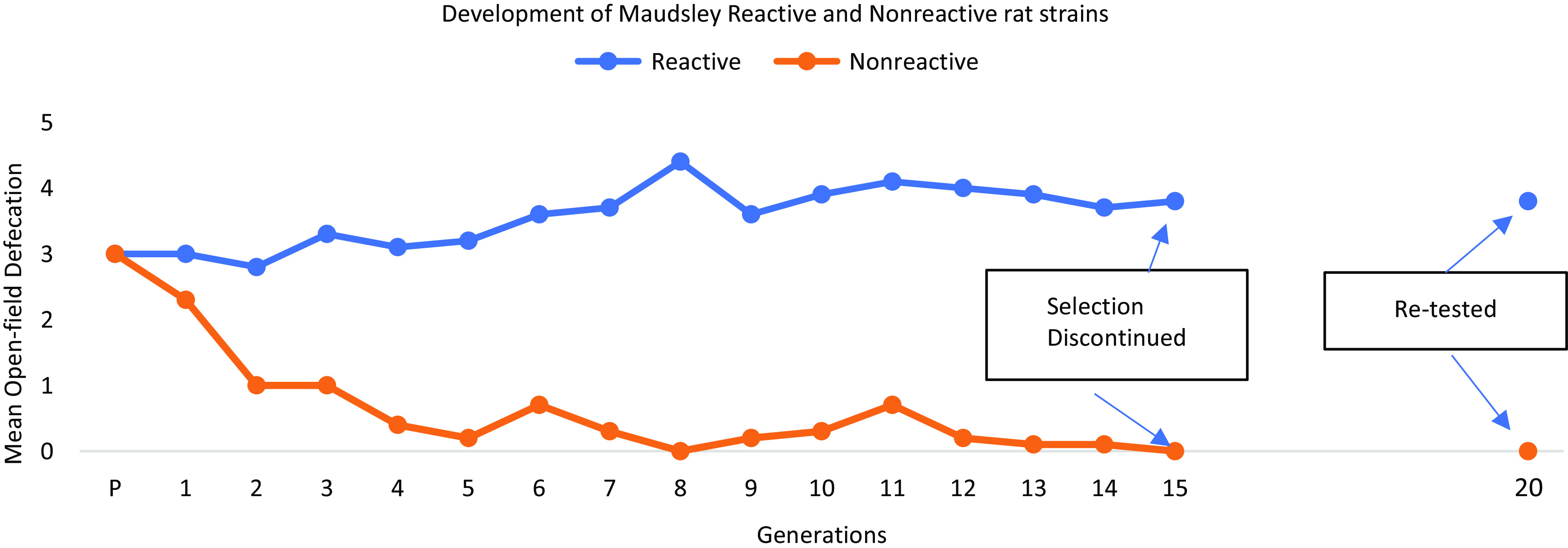
Redrawn from Broadhurst (1960). The effects of genetic selection combined with inbreeding on open-field defecation scores in the Maudsley Reactive and Nonreactive rat strains. Selection was discontinued at Generation 15 and animals were retested in Generation 20. The phenotypic differences between the strains were maintained.

## 1. Development of the strains; propagation of stocks

Broadhurst (1960) developed the Maudsley strains at the Animal Psychology Laboratory of the University of London’s Institute of Psychiatry in Beckenham, Kent, following an earlier successful genetic selection experiment by Calvin Hall, initiated in the 1930s (described in Hall, 1951). Broadhurst selected for high and low defecation in a brightly illuminated arena accompanied by white noise as an acoustic stimulus by breeding male and female rats with the highest OFD scores (MR strain) and, conversely, those with the lowest scores (MNR strain) for 15 generations. After discontinuing selection at Generation 15, he retested the strains at Generation 20 and found the differences in OFD had been maintained (Broadhurst, 1962). The strains have been inbred by brother/sister mating to fix key loci in the homozygous state. It was noteworthy that the primary deviation from the foundation population mean OFD score was in the direction of *decreased* defecation and that it was achieved within a few generations. Analyses of data for the first five generations of MNR rats could not reject the hypothesis of involvement of a major gene in the OFD response to selection (Liang, 1978, personal communication). The strains were exported to the National Institutes of Health in Bethesda, Maryland, and to the University of Northern Iowa (Harrington, 1981). Responding to concern that OFD scores of MNR rats of the North American stocks had increased (Harrington, 1972), strain differences in OFD were found to resemble those typical of English stocks when they were maintained under similar conditions to those in use at the Animal Psychology Laboratory in England (Harrington & Blizard 1983), a result consistent with the idea that the genetic variants underlying OFD in the strains were fixed. Cryopreserved embryos from sublines of the strains (MR/Har/Bliz and MNRA/Har/Bliz and from the MR/N strain from the former NIH colony) are kept at the University of Missouri Rat Resource Center in Columbia, Missouri (https://www.rrrc.us), and can be rederived as necessary.

## 2. Validation of selection criterion

Broadhurst relied on Calvin Hall’s earlier attempts (Hall, 1934, 1951) to validate OFD as a measure of emotionality and provided additional support of his own in unselected Wistar rats (Broadhurst, 1957a, 1957b, 1958). In humans, gastrointestinal (GI) discomfort (both diarrhea and constipation) accompanies anxiety and stress. Broadhurst also chose to cite the excretory reactions of humans in warfare (Stouffer et al., 1949) to support the relevance of this physiological system to human stress response (Broadhurst, 1960). Thus, OFD in rodents as a measure of mammalian reactivity to stress is plausible both from the perspective of experimental approaches in laboratory rats and from observations in humans during everyday life and when under extreme stress.

Cross-species relevance of elements of the open-field test should be considered in evaluating its appropriateness for the development of a model system. The very bright illumination in the open-field test is a plausible stressor when considering the photophobic nature of the rat but response to bright lights is not at the forefront of discussions of human N. More generally, Archer (1973, 1975) argued that, rather than representing a unitary dimension (as conceptualized in humans), rat emotionality is better conceived as a range of expressive behavioral patterns displayed in a situation-specific manner. From this perspective, selection on a single criterion such as OFD might not capture the full range of emotionality in rats.

## 3. Behavioral and neurochemical differences in strains

Two reviews summarized the many behavioral comparisons of the strains that were conducted in England and suggested that the results were consistent with the idea that MR rats have greater stress reactivity (see later discussion of this characterization) than MNR (see tabular presentation of these results in Broadhurst, 1975; Eysenck & Broadhurst, 1964). Later behavioral research (reviewed in Blizard & Adams, 2002) tested the strains in social contexts including agonistic behavior. Resident MR males were observed attacking intruders introduced into a home cage or a small colony and attacking familiar colony mates more often than MNR males across these settings. Differences in social behavior between the strains may emerge in early life: when weanlings were tested for emergence from their nest box while either a strange male or the rat’s mother was confined at the center of their home cage, MRs of both sexes were quicker to emerge and were more active near the strange male compared to MNRs, whereas both strains uniformly emerged and made contact when their mother was the stimulus animal. This latter test was unique in presenting a social test stimulus in a familiar setting.

Blizard (1981, 1989) and Blizard and Adams (2002) reviewed later research of primarily North American stocks, focusing more on the physiological and neurochemical basis of emotionality as represented in the central and peripheral sympathetic nervous system. In part, these findings cohere with the idea that genetic selection for OFD in the Maudsley model has affected both the central noradrenergic system where MNRs exhibit greater *sustained* cerulear response to chronic stress (Blizard, Freedman, & Liang, 1983) and the peripheral noradrenergic system where MNRs possess increased sympathetic tone in many organs (Blizard, Altman, & Freedman, 1982; Liang & Blizard, 1978; Slater, Blizard, & Pohorecky, 1977).

## 4. Interpretive issues underlying behavioral comparisons

The large number of behavioral comparisons of the strains referred to above consisted, for the most part, of screening the two strains on standard behavioral tests without exploration of the specific mechanisms as to why strain differences occurred. For example, MNR rats were found to perform escape avoidance conditioning more efficiently than MR rats, and this was interpreted to imply that the putatively greater fear/emotionality of MR rats impaired their acquisition of the task. Nevertheless, protocols used for avoidance conditioning vary widely: for example, shock intensity imposed, nature of the conditioned stimulus, including its duration, physical parameters inside the conditioning chamber, etc., can interact with characteristics of the tested subjects to enhance or obscure group differences. If the Maudsley strains differed in pain thresholds, or in activity levels in the chambers before conditioning trials commenced, would such differences affect interpretation of any strain differences that emerged during conditioning? This concern is especially cogent given the lack of replication of the strain difference in active avoidance conditioning by Harrington (1979) and others (for discussion, see Blizard and Adams, 2002).

Pertinent questions can be asked of many of the other tests in which MR/MNR differences were found. More generally, the use of electric shock in several of the relevant test situations introduced a stimulus that is not ecologically relevant to the rat. To a large extent, this concern is met by introducing social stressors as a complement to or even as a substitute for some of the existing traditional stress stimuli (Adams & Blizard, 1987; Blanchard et al., 1994; Martinez, Calvo-Torrent, & Pico-Alfonso, 1998). Clearly, there is face validity for evolutionarily meaningful social contexts where defeat or low social status may correspond to lowering of reproductive success (Adams & Boice, 1983; Blanchard, McKittrick, & Blanchard, 2001).

Differences in stress reactivity were invoked earlier to account for differences between the strains. Activation of the hypothalamic-pitutitary-adrenal (HPA) axis is commonly used as an index of stress, and the Maudsley strains have not been found to differ in adrenocorticotropic hormone (ACTH)/corticosterone response to painful stimuli (Blizard, Eldridge, & Jones, 2015). It would be helpful to characterize the stress dimension more specifically when considering behavioral outcomes so as to be able to predict the direction of differences between groups before a test is administered rather than in a post hoc manner.

Aside from the results of standardized behavioral testing, a notable difference between the strains was observed during routine handling; MNR rats were more tractable, had lower muscle tone, and were flaccid, often hanging limply when held gently by the shoulders and neck. In contrast, MRs tended to struggle and resist handling (Blizard, personal communication, 1990).

## 5. Peripheral sympathetic nervous system and emotionality

Research conducted on North American stocks of the Maudsley rats showed that, under resting conditions, MNR rats had substantially higher concentrations of norepinephrine (NE) in peripheral organs than MR rats (e.g., Blizard, et al., 1982; Liang & Blizard, 1978; Slater et al., 1977), and one interpretation of these differences is that MNR rats’ organs are under tonic peripheral sympathetic stimulation. A corollary of this finding is that, when stressed, there is the potential for greater efflux of NE onto organs of MNR rats. In the colon, such an event would have the potential to relax smooth muscle and inhibit colonic motility and is consistent with MNR rats’ behavior when placed in the open-field test (defecation scores are effectively zero). Additional strain differences in GI processing have been reported, which must be considered in developing an appropriate understanding of how genetic selection has impacted this system in the strains. For example, following mild food deprivation, after being fed a small meal in their home cage, MNR rats excrete more fecal boli than MR rats, the opposite of the strain difference that occurs in the open-field test (Blizard, 1989, personal communication). Thus, neurological or neurochemical systems favoring increased GI and/or colonic motility under basal conditions may have also been selected in MNR rats. Furthermore, the cited changes in the peripheral sympathetic nervous system, brought about by genetic selection, have the potential to account for strain differences in emotionality without positing primary variations in the CNS.

Another incidental observation was that the eyes of the MNR rats were much more prominent than those of MR rats, a larger amount of the eyeball could be seen (Blizard, 1987, personal communication). If this was the result of sympathetic overstimulation of upper eyelid retractor muscles, as is typical in patients suffering from Graves disease, it would be consistent with the increased sympathetic tone in MNR rats noted above.

## 6. Central noradrenergic system and emotionality

MNR rats showed greater *sustained* tyrosine hydroxylase elevation in the locus ceruleus (LC), following chronic stress than MR rats (Blizard et al., 1983), and opened the possibility that an important central noradrenergic nucleus might have been the focus of genetic selection (Blizard, 1988). Intriguingly, an earlier attempt to explore the neurological basis of anxiety (Gray & McNaughton, 2000) emphasized the potential role of the dorsal noradrenergic bundle (DANB) in the medial septal-hippocampal inhibition system in anxiety. Stimulation of hippocampal theta rhythm from electrodes implanted in the medial septum has a frequency-specific relationship to current driving intensity, with a minimum at 7.7 Hz in outbred male rats, which is lacking in putatively less emotional female rats and MNR rats. MRs, on the other hand, resemble unselected outbred rats with a minimum at 7.7 Hz. Administration of antianxiety drugs and depletion of DANB noradrenaline content by neurochemically specific toxins also abolished the frequency-specific minimum at 7.7 Hz in unselected rats. The DANB originates in the LC, so the discovery of differences in the magnitude of strain-specific changes in response to chronic stress in this locus in the Maudsley model is an intriguing correlation that draws attention to the need for further examination of the central noradrenergic system in these strains. The LC projects to many areas of the forebrain and group differences in its biosynthetic capacity may have important functional implications in its terminal regions. Understanding of the LC and its role in neurophysiological and behavioral response has changed considerably since the findings described above were reported. At that time, an idea promoted by Amaral and Sinnamon (1977), that activation of the LC improved signal-to-noise ratio in its terminal regions, was influential. On the other hand, this was a hypothesis relevant to the neurophysiological dimension and was not necessarily easy to translate into specific behavioral outcomes. More recent approaches (Poe et al., 2020) emphasize the discrete role of ascending projections from the LC, and none of these have been related to the manifold behavioral differences between the two strains. Later, we discuss recent work on the role of descending projections of the LC on colonic function.

## 7. Human colon function and rodent models

As noted, the differences in OFD between the Maudsley strains are associated with variation in colonic function under resting conditions and correlated with alterations in the peripheral sympathetic nervous system. IBS in humans represents a variety of functional GI disorders in which stress and personality factors have often been implicated, and exploration of mechanisms underlying these associations has revealed new insights into a biological mechanism via which genetic selection for OFD may have been achieved. Recently, Kurahashi’s laboratory (Kurahashi et al., 2020a, 2020b) has provided evidence of the existence of two α_1_ adrenergic receptors (α_1A_ and α_1D_) in mouse and human colon, which have opposing effects on colonic muscle. Specifically, α_1D_ receptors located on smooth muscle cells (SMC) of mouse and human colon resulted in contraction of colonic muscle when exposed to *1 µm* NE, while α_1A_ receptors, located on PDGFRα+ cells, inhibited SMC cells via the AR-SK signaling pathway when exposed to *10 µm* NE. Alpha_1_ receptors in rat colon could represent the substrate upon which the altered levels of NE existing in colonic tissues (Blizard et al., 1982) of the two strains could exert their effect. Thus, the higher levels of NE in tissues of MNR rats released onto α_1A_ receptors in the colon during stress could diminish the amplitude of colonic contractions and reduce or prevent OFD. Conversely, in nonstress situations, such as the home cage, release of lower or basal levels of NE could stimulate colonic contractility via α_1D_ receptors and result in the higher fecal output following a meal in MNR rats (described earlier). The suggested role of α_1_ receptors in human and mouse colon is a novel finding discovered by investigators chiefly interested in IBS. We hypothesize that alterations in sympathetic tone, such as we have suggested to exist in the Maudsley model, could interact with these receptors to produce these functional disorders. Aside from presynaptic influences, the possibility exists that genetic selection for OFD may have also assorted different densities of colonic α_1_ receptors or different colonic receptor types in the two strains.

Earlier, differences in the function of the LC between the Maudsley strains were discussed in relation to their potential impact on the CNS and their role in strain differences in behavior. New insight into the role of *descending* projections of the LC on autonomic function has recently been reported. Kong et al. (2023), studying C57BL/6J mice, found that stimulation of a projection of the LC to the rostral ventromedial medulla has an hyperalgesic effect on nociceptive input from the descending colon and rectum. Previous research had shown that stimulation of direct projections from the LC to the dorsal horn had an opposite analgesic effect in response to colorectal distention in rats (Liu, Tsuruoka, Maeda, Hayashi, & Inoue, 2007). These results show that the LC plays an important role in influencing afferent information from a key organ (colon) in GI processing. More generally, the discovery of important relationships between the LC and the colon provides an important focus for exploration of the relationship between the central and peripheral nervous systems in the Maudsley model.

## 8. IBS, personality, and genetics

In human genetic studies, there is a developing literature on the relationship of IBS to personality in which the contribution of genetics has been examined: Eijsbouts et al. (2021) conducted genome-wide analyses of 40 548 people with IBS from the UK Biobank and 12 852 from the Bellygenes initiative (a worldwide study aiming to identify genes linked to IBS) and compared them to 433 201 controls from the UK Biobank. They identified six susceptibility loci, which were replicated in a 23andMe panel of approximately four times as many cases and controls. Four of the loci were also associated with mood and anxiety disorders or were expressed in the nervous system. Specifically, there were significant genome-wide correlations between risk of IBS, anxiety, N, and depression of 0.5 or higher. In contrast, genetic correlations with other psychiatric disorders were substantially lower. The predominant conception of the relationship between IBS and anxiety is that the latter “causes” abdominal symptoms. However, Eijsbouts et al. conducted additional analyses to explore the role of shared genetic risk versus other conceptual models. They removed participants with anxiety from the IBS GWAS and removed participants with IBS from the GWAS for anxiety. The genetic correlation between IBS and anxiety remained and was estimated at 0.31 (SE = 0.06). Bidirectional Mendelian randomization and other analyses also showed that anxiety or depression and IBS are the results of shared etiologic pathways rather than one causing the other. Applying the same logic to the Maudsley model, it is possible that any influence of genetic selection on brain and behavior reflects the effect of the same genes that altered the peripheral sympathetic nervous system, not because one caused the other, as implied by the choice of the selection criterion, but because they are separate outcomes of the same neural pathways acting in the brain and the periphery.

Future research on IBS focused on specific mechanisms, regardless of their location in the body, will have to be conducted in a manner that recognizes the strongly held view (Mayer, Ryu, & Bhatt, 2023) that this functional disorder must be investigated and treated using a multidimensional approach that encompasses multiple biological mechanisms as well as attention to environmental factors.

## 9. Heritability of neuroticism trait in humans

Large-scale studies of the N dimension have provided more precise estimates of its heritability. N is a heritable trait in humans with a broad-sense heritability of 48% based on a meta-analysis of six twin cohorts (total N 29 496 twin pairs; van den Berg et al., 2014) and confirmed by pedigree analyses (Boomsma et al., 2018) as well as literature reviews (Sanchez-Roige, Gray, MacKillop, Chen, & Palmer, 2017). Vukasović and Bratko (2015) noted the higher heritability estimates in twin studies (47%) compared to family and adoption studies (22%) and attributed these differences to nonadditive genetic effects, which contribute to resemblances of twins and full siblings, but not to resemblance of nearly all other relatives.

Large-scale studies have also enhanced our understanding of the N dimension by providing analyses of its relationship to other personality dimensions, based on GWAS results. Exemplary of this trend are the findings of a comprehensive analysis which examined the relationship of the Big Five personality dimensions to loneliness (Abdellaoui et al., 2019a) in more than 29 000 twins and their family members. All personality traits were correlated with loneliness, but only N showed a significant relationship to loneliness (*r* = 0.50) after correcting for the remaining four personality traits. Loneliness has an estimated heritability of 42% (Distel et al., 2010). In the study of Abdellaoui et al. (2019a), single-nucleotide polymorphisms (SNPs) data were available in ∼4000 subjects. From the molecular data, it was estimated that the SNP (i.e., narrow-sense) heritability was 22% for N and 14% for loneliness. Narrow-sense heritability, of course, is particularly important when considering selection experiments because it is the magnitude of the narrow-sense heritability that gauges the additive genetic variance which selection can assort. A genetic correlation between these traits was estimated at .71, and second larger study (Abdellaoui et al., 2019b) confirmed this estimate (*r*_*g*_ = 0.69). N’s correlation with loneliness draws attention to the social milieu for the expression of this dimension and underlines the potential significance of studies of social behavior in the Maudsley strains referred to earlier.

## 10. GWAS studies of neuroticism

**Figure 2.**
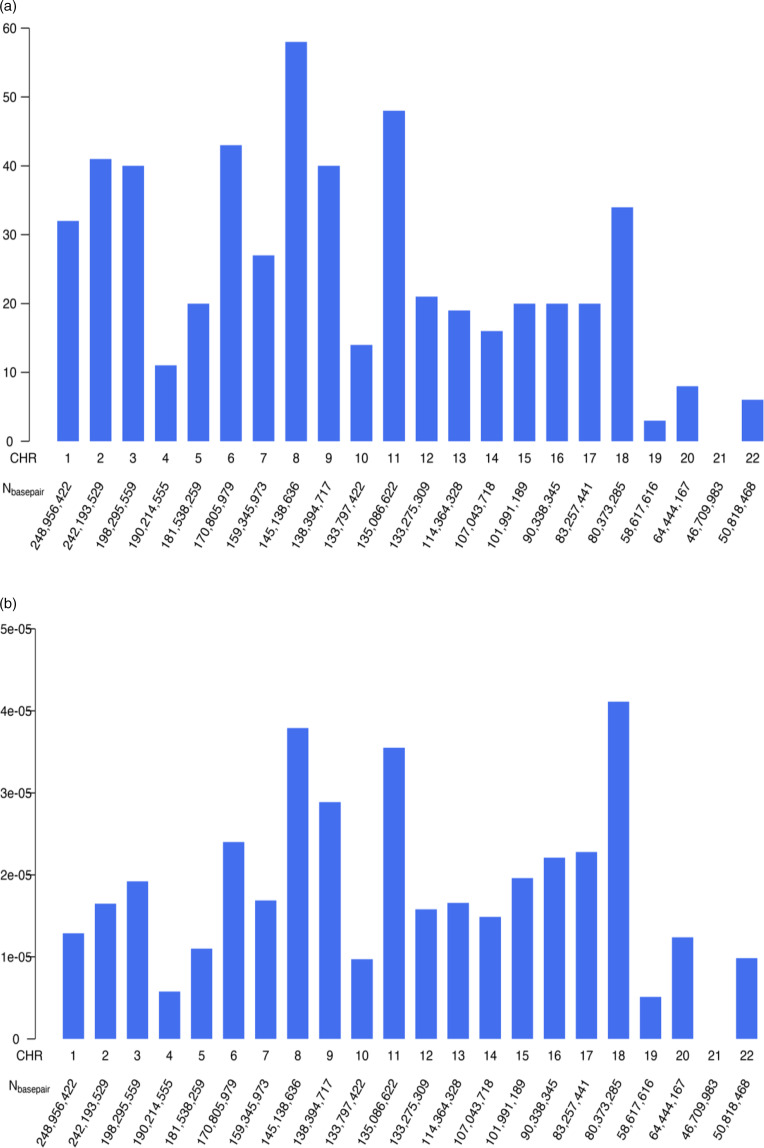
Based on Supplement Table 3 in van der Walt et al. (2023). X-axis: chromosome number and length of chromosome (number of base pairs, corresponding to https://www.ncbi.nlm.nih.gov/grc/human/data). Y-axis: 2A: number of hits on each chromosome is a sum of hits on each chr from Sup Table 3. 2B: proportion of hits per chromosome.

Research in humans has led to multiple discoveries of regions in the genome that are implicated in N. Inspired initially by the seminal paper by Flint et al. (1995) that demonstrated the feasibility of identifying quantitative trait loci (QTLs; regions in the genome influencing quantitative phenotypes or traits) in mice for complex traits such as anxiety, linkage studies of N were undertaken (e.g., Wray, Kemper, Hayes, Goddard, & Visscher, 2008), as well as candidate gene studies that, for example, exploited the known synteny between mouse and human loci to screen the human genome for loci identified in mice (Fullerton et al., 2008). The initial enthusiasm for linkage studies in humans diminished rapidly when power simulations for complex traits were carried out based on realistic effect sizes. Likewise, candidate gene studies did not prove a fruitful approach. For example, QTLs for major depressive disorder discovered in genome-wide studies do not generally confirm the significance of candidate genes (e.g., Bosker et al., 2011). Breakthroughs in genotyping technology enabled screening large numbers of participants for SNPs and genome-wide association studies became feasible, leading to several projects on N. Van der Walt, Campbell, Stein, and Dalvie (2023) identified 32 GWASs of anxiety disorders, nondiagnostic anxiety traits, and N that reported 563 independently significant variants, with 29 replicated nominally in independent samples and three replicated significantly. In considering the low replication rate, van der Walt et al. reached the sobering conclusion that future GWAS investigations would need to increase sample sizes into the millions. We took their supplementary report for N based on eight meta-analyses of GWAS studies, the first one published in 2015 and the most recent one in 2019 (Baselmans et al., 2019; de Moor et al., 2015; Kim et al., 2017; Lo et al., 2017; Luciano et al., 2018; Nagel et al., 2018; Okbay et al., 2016; Smith et al., 2016). Figure 2 summarizes these genome-wide significant results for N and plotted the total number of hits for each chromosome (Fig. 2A) and the proportion of hits per chromosome (Fig. 2B). Generally, the larger chromosomes (chromosome length is included under the X-axis) tend to have a larger number of significant hits, with some notable exceptions for chromosomes 8 and 18. The genes coding for the receptors relevant to contraction and relaxation of colonic muscle mentioned earlier do not align with any of the GWAS regions in Figure 2B.

## 11. Developments in rat behavioral genetics

When Broadhurst conducted his selection experiment, the laboratory rat was the animal of choice in most psychology departments. During the course of the molecular revolution, most studies focused on the mouse because it was the traditional organism of choice for mammalian genetic studies, and investigators interested in behavior genetics accordingly switched their focus to that species. Nevertheless, the enormous literature on rat behavior remains a huge resource and has stimulated developments in rat genetics. In one mapping study, investigators studied F2 offspring of a cross between two rat lines selected for differences in sensation-seeking behavior. Relevant to the present interest in defecation response, they identified a statistically significant locus on rat chromosome 18 for frequency of defecation in the open field (Chitre et al., 2022). Another study (Munro et al., 2022) has mapped regulatory genes affecting expression of genes in five brain regions using genetically heterogeneous rats derived from a cross of eight strains originating in the former NIH colony. These results from the Palmer laboratory are available online at RatGTEx.org where expression data for individual genes as well as expression QTLs can be accessed. These developments in genetics will be an important resource for future investigations of rat models of human personality dimensions.

## 12. Conclusions

After its initial conception and development, the Maudsley model became an object of fascination in its own right. More and more comparisons were made between the strains, each one appearing to add to the presumptive validity of the model. On the other hand, the findings were seldom held up as a window to elucidate the human dimension of N. Obviously, disciplinary specialization made this difficult for both animal and human researchers. It is now time to use the model for its original purpose. In this brief review, we have tried to show that a bidirectional process of exchange between the animal model and the human dimension of N can be productive. We have focused attention on the relationship between the respective roles of the central and peripheral nervous systems in emotional behavior in the animal model and raised questions about simplistic notions of cause and effect that can be fruitfully applied when considering the N dimension. Seeing phenomena through a genetic lens also provides an excellent means of promoting animal/human exchanges, and this is facilitated by the extraordinary advances in understanding and analysis of the mammalian genome. This process of exchange needs to be strengthened (see also e.g., Cacioppo et al., 2015). Some exciting developments also include genetic prediction across species (Wray et al., 2019). This issue of *Personality Neuroscience* is an important attempt to promote this kind of interaction so that future progress is achieved via a more inclusive process.
